# The Roles of Peyer's Patches and Microfold Cells in the Gut Immune System: Relevance to Autoimmune Diseases

**DOI:** 10.3389/fimmu.2019.02345

**Published:** 2019-10-09

**Authors:** Nobuhide Kobayashi, Daisuke Takahashi, Shunsuke Takano, Shunsuke Kimura, Koji Hase

**Affiliations:** ^1^Division of Biochemistry, Faculty of Pharmacy and Graduate School of Pharmaceutical Science, Keio University, Tokyo, Japan; ^2^Department of Bacteriology, Graduate School of Medical Sciences, Kanazawa University, Kanazawa, Japan; ^3^International Research and Development Center for Mucosal Vaccines, The Institute of Medical Science, The University of Tokyo (IMSUT), Tokyo, Japan

**Keywords:** mucosal immunity, autoimmune disease, microfold cell, Peyer's patch, intestinal microbiota, intestinal epithelial cell

## Abstract

Microfold (M) cells are located in the epithelium covering mucosa-associated lymphoid tissues, such as the Peyer's patches (PPs) of the small intestine. M cells actively transport luminal antigens to the underlying lymphoid follicles to initiate an immune response. The molecular machinery of M-cell differentiation and function has been vigorously investigated over the last decade. Studies have shed light on the role of M cells in the mucosal immune system and have revealed that antigen uptake by M cells contributes to not only mucosal but also systemic immune responses. However, M-cell studies usually focus on infectious diseases; the contribution of M cells to autoimmune diseases has remained largely unexplored. Accumulating evidence suggests that dysbiosis of the intestinal microbiota is implicated in multiple systemic diseases, including autoimmune diseases. This implies that the uptake of microorganisms by M cells in PPs may play a role in the pathogenesis of autoimmune diseases. We provide an outline of the current understanding of M-cell biology and subsequently discuss the potential contribution of M cells and PPs to the induction of systemic autoimmunity, beyond the mucosal immune response.

## Introduction

The mucosal surface forms a border between our body and the outer world. The human intestinal mucosa is exposed to food-derived antigens, pathogens, and more than 40 trillion commensal bacteria (1). The mucosal surface is protected from invasion of foreign antigens by efficient and size-selective physical shields composed of mucin and glycocalyx layers and chemical barriers including antimicrobials and antigen-specific secretory immunoglobulin (Ig) A (S-IgA) (2). In vertebrates, the S-IgA response is produced in gut-associated lymphoid tissues (GALTs), one of the largest immune response-inductive sites in the body (3). Organized GALTs are composed of several lymphoid tissues, such as Peyer's patches (PPs) of the small intestine, cecal patches, colonic patches, and isolated lymphoid follicles, present throughout the gastrointestinal tract. GALTs are dedicated to sampling and inducing adaptive immune responses against potentially hostile foreign agents as well as non-harmful commensal microorganisms via several mechanisms. For instance, CX_3_CR1^+^ mononuclear phagocytes that reside in the intestinal lamina propria directly sample luminal bacteria by forming transepithelial dendrites (TEDs). Dendrite protrusion by CX_3_CR1^+^ cells is regulated by the pyruvate/lactate-Gpr31 axis (4). Goblet cell-associated antigen passages (GAPs) form another antigen sampling machinery by transporting soluble antigens from the lumen to the lamina propria. TEDs and GAPs are observed in villi to transport antigens to the lamina propria, but not to organized GALTs such as PPs. TEDs and GAPs are discussed in detail elsewhere (5, 6).

**Figure 1:**
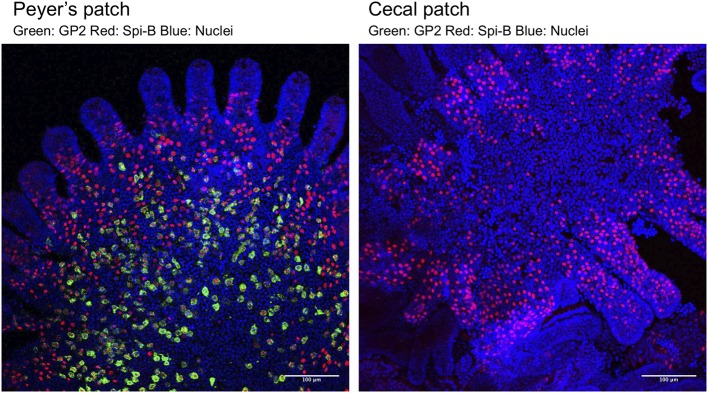
Whole-mount immunostaining of M cells in FAE of a PP **(Left)** and a cecal patch **(Right)**. M cells were classified into two populations based on the expression of the molecular markers Spi-B (red) and GP2 (green). Spi-B^+^ single-positive cells are immature M cells located near the crypt and have low ability to take up luminal antigens. Spi-B^+^GP2^+^ double-positive cells are functionally mature M cells that are located near the top of the FAE dome of PP and have high uptake capacity. In cecal patches, Spi-B^+^GP2^+^ double-positive cells are rarely detected. See Kimura et al. (8) for details.

Specialized epithelial microfold (M) cells located in the follicle-associated epithelium (FAE) are responsible for antigen uptake in GALTs and nasopharynx-associated lymphoid tissue and thus play a central role in immune surveillance on the mucosal surface (7) (Figure 1). M cells recognize luminal antigens by expressing cell-surface receptors and actively engulf these antigens at their apical surface and then exocytose them through their basolateral plasma membrane, a process termed transcytosis. Transcytosis facilitates the delivery of luminal antigens to mononuclear phagocytes, such as dendritic cells (DCs) and macrophages, and B cells localized at the subepithelial dome (SED) of PPs to trigger antigen-specific immune responses, such as antigen-specific S-IgA production (9). Given that S-IgA plays a vital role in protection against pathogens as well as the establishment of a mutual relationship with the gut microbial community, M cell-mediated antigen uptake potentially contributes to the maintenance of gut immune homeostasis. However, various pathogenic agents, such as *Salmonella, Brucella*, botulinum toxin, and prions exploit M cells as a portal for invasion (Table 1). Hence, M cells have a bilateral character: they are key for immunosurveillance and represent a gate for pathogens in the mucosa. In the small intestine, PPs are the most important sites for the induction of T cell-dependent IgA class switch recombination (3). The human small intestine at young-adult age harbors more than 200 PPs, and nearly half of these are concentrated in the distal 25 cm of ileum, whereas the mouse small intestine possesses 8–12 PPs (depending on the strain) located in the proximal and distal regions (10, 11). While peripheral lymph nodes drain lymphatics, PPs lack afferent lymphatic vessels and instead are equipped with the luminal antigen sampling machinery represented by M cell-dependent transcytosis. Moreover, different from peripheral lymph nodes, which lack obvious germinal center (GC) responses under physiological conditions, PPs constitutively form GCs in response to commensal microorganisms and food-derived antigens taken up by M cells (3). GC formation is a prerequisite for somatic hypermutation and affinity maturation of IgA class-switched B cells in PPs. Although M cell-dependent antigen uptake is dispensable for the initiation of PP development, it plays a critical role in the maturation of PPs. Indeed, M cell-deficient mice have smaller B-cell follicles, reduced GC reaction, and a lower level of IgA production (12, 13). Thus, M cells are critical for the development and function of mucosal IgA responses.

**Table 1:** Microorganisms transported by M cells.

**Microorganisms**	**Host analyzed**	**Ligand**	**Receptor**	**Microorganisms**	**Host analyzed**	**Ligand**	**Receptor**
**Bacteria**				**Viruses**			
*Bacillus* Calmette-Guérin (BCG)	Rabbit	–	–	Human immunodeficiency virus type 1 (HIV-1)	Mouse, rabbit	–	–
*Brucella abortus*	Calf, mouse	Hsp60	PrP^C^	Mouse mammary tumor virus (MMTV)	Mouse	–	–
*Campylobacter jejuni*	Rabbit	–	–	Norovirus	Mouse	–	–
*Escherichia coli* K12	Mouse	FimH	GP2	Poliovirus type 1	Human	–	CD155
*Escherichia coli* RDEC-1 (rabbit EPEC)	Rabbit	–	–	Reovirus type 1 and type 3	Mouse	Protein σ1	α2, 3-linked sialic acid
*Escherichia coli O:124 K:72*	Rabbit	–	–				
Group A *Streptococcus* (GAS)	Mouse	–	–	**Parasites**			
*Lactobacillus acidophilus* L-92	Mouse	SlpA	Umod	*Cryptosporidium*	Guinea pig	–	–
*Mycobacterium avium* subsp*. Paratuberculosis*	Goat	–	–	*Eimeria coecicola*	Rabbit	–	–
*Mycobacterium tuberculosis*	Mouse	–	–				
*Salmonella enteritidis*	Rabbit	–	–	**Prion**			
*Salmonella typhi*	Mouse	–	–	PrP^Sc^	Mouse	–	–
*Salmonella typhimurium*	Mouse	FimH	GP2				
*Shigella flexneri*	Rabbit	–	–	**Toxin**			
*Streptococcus pneumonia* R36a	Rabbit	–	–	Botulinum toxin A complex (L-PTC)	Mouse	HA	GP2
*Streptococcus pyogenes*	Rabbit	–	–				
*Vibrio cholerae*	Rabbit	–	–				
*Yersinia enterocolitica*	Mouse	Invasin	β1 integrin				

Accumulating studies have revealed that disturbance of the gut microbial community (termed dysbiosis) as well as the expansion of specific bacterial species are implicated in the development of multiple systemic diseases, including autoimmune diseases (14–18). Molecular mimicry of epitopes between host proteins and bacterial antigens might be a causative factor in certain autoimmune diseases, such as Guillain–Barré syndrome (GBS) (19). Immune responses in PP to particular commensal bacteria are required for the development of autoimmune disease in a mouse model (20). These bacteria or their components may penetrate the epithelial barrier to induce autoimmune responses; however, the underlying pathological mechanisms are yet to be elucidated. A recent study demonstrated that M cell-dependent antigen uptake might facilitate not only mucosal but also systemic immune responses, such as antigen-specific IgG production (9). This suggests that antigen uptake by M cells might play a pathological role in the production of autoantibodies and the development of autoimmune diseases. In this review, we discuss recent advances in the molecular basis of differentiation and functions of M cells and the potential implication of GALT in the etiology of various autoimmune diseases.

## Intestinal Epithelial Cells Constituting the Mucosal Barrier

Intestinal epithelial cells serve as a barrier that segregates the internal milieu from luminal components, including commensal microbiota and food antigens. All intestinal epithelial cell lineages are derived from intestinal stem cells (ISCs) located at crypt bottoms (21). ISCs differentiate into functionally mature epithelial cells via actively proliferating transient amplifying cells. In the small intestine, ISCs are surrounded by Paneth cells that secrete antimicrobial peptides to prevent the raid on crypt bottoms by microbes (22). The antimicrobial peptides secreted by Paneth cells establish the chemical barrier to protect intestinal tissue against the bacterial invasion. Paneth cells also constitute the niche for ISCs by providing proteins essential to maintaining plasticity (23). Goblet cells secrete the glycoprotein mucin, the main component of the mucous layer (24), which prevents the adhesion of bacteria on epithelial cells. Enteroendocrine cells express chemoreceptors on their apical side to detect luminal substances (25). They secrete gastrointestinal hormones to promote the secretion of digestive juice and stimulate gastrointestinal motility. Tuft cells are essential for the protective immunity against parasitic helminths. Interleukin (IL)-25 production by tuft cells activates tissue-resident group 2 innate lymphoid cells (ILC2s) (26), which then secrete IL-13 to promote the self-renewal of ISCs and the differentiation of goblet and tuft cells (26, 27). Collectively, these epithelial cell lineages mainly work as the barrier to prevent the penetration of foreign antigens. In contrast, M cells are specialized for the uptake of luminal antigens, including live bacteria.

## Molecular Mechanism of M-Cell Differentiation

**Figure 2:**
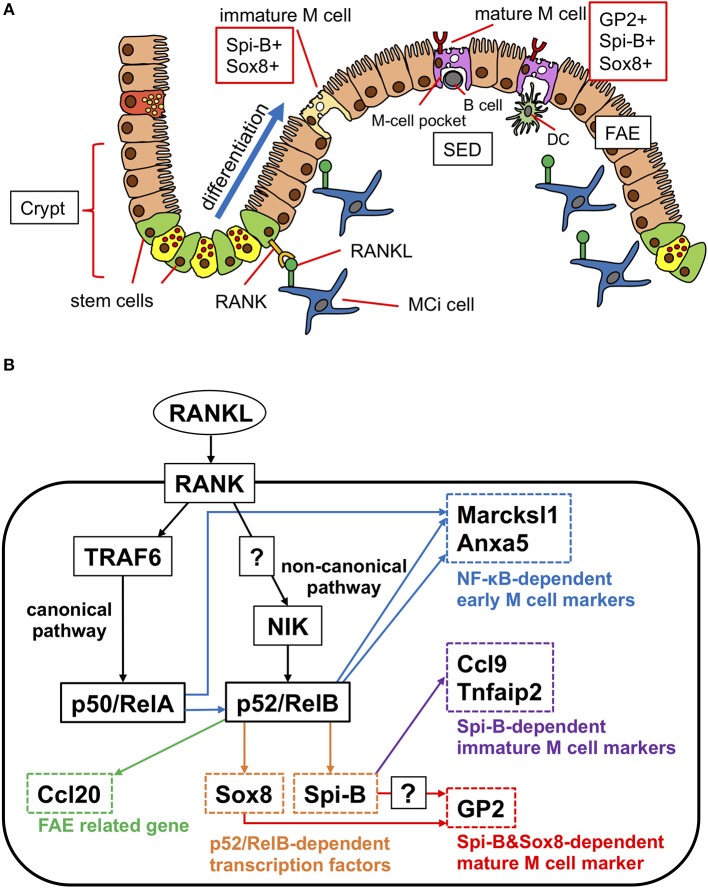
Scheme of differentiation of M cells in PP. **(A)** RANKL expressed on M-cell inducer (MCi) cells initiate the differentiation of M cells. RANK signaling induces the transcription factor Spi-B and Sox8 parallelly. Spi-B^+^Sox8^+^GP2^−^ cells are immature M cells that have low uptake activity. Sox8 directly induces the expression of GP2. GP2^+^ cells are mature M cells that have high uptake activity. **(B)** Signaling pathway of M-cell differentiation. RANKL signaling activates canonical NF-κB (p50/RelA) through TRAF6. Canonical NF-κB signaling is required for the activation of non-canonical NF-κB (p52/RelB), which regulates early M-cell marker genes. p52/RelB directly upregulates the expression of transcription factors Spi-B and Sox8. Sox8 directly binds to the *Gp2* promoter. Both Spi-B and Sox8 are required for *Gp2* expression and M-cell maturation.

Like other intestinal epithelial cells, M cells are generated from Lgr5^+^ ISCs (28) (Figure 2A). At the early stage of differentiation, M-cell precursors upregulate Marcksl1 and Anxa1, and in turn, functionally and morphologically immature Spi-B^+^ glycoprotein 2 (GP2)^−^ M cells are generated (Figure 2B). The immature M cells terminally differentiate into Spi-B^+^ GP2^+^ mature M cells with high uptake activity (8). This process is initiated by exposure to receptor activator of nuclear factor κB (NF-κB) ligand (RANKL), which is abundantly produced in the SED region of GALT (12) (Figure 2A). Subepithelial mesenchymal cells highly expressing the membrane-bound form of RANKL have been recently defined as M-cell inducer cells (13). Selective deletion of membrane-bound RANKL in M-cell inducer cells abolished the development of M cells in FAE. RANKL binds with its receptor RANK on FAE to activate NF-κB-inducing kinase (NIK) and the downstream non-canonical NF-κB pathway (8, 29). Eventually, a heterodimer of the transcription factors RelB and p52 is translocated into the nucleus to upregulate M-cell signature genes, including *Spib*, a member of the E-26 (ETS) transcription factor family (8) (Figure 2B). As mice lacking epithelial NIK are defective in M-cell development, the non-canonical NF-κB pathway is indispensable for M-cell differentiation (29).

In addition to the non-canonical NF-κB pathway, tumor necrosis factor (TNF) receptor-associated factor 6 (TRAF6), the adaptor protein essential for activation of the canonical NF-κB pathway, is necessary for the differentiation of M cells (30) (Figure 2B). In intestinal organoids, RANKL treatment upregulates several M-cell marker molecules including GP2; however, the M cell-inducing effect of RANKL is canceled by inhibition of the canonical NF-κB pathway. Nevertheless, forced expression of p50/RelA, canonical NF-κB transcription factors downstream of TRAF6 (Figure 2B), in intestinal organoids upregulates only early and middle M-cell marker genes, such as *Marcksl1*, with *Spib* being expressed to a lesser extent (30). Importantly, p50/RelA also increases the expression of RelB and p52, indicating that the canonical NF-κB pathway is not sufficient for full differentiation of M cells but may indirectly facilitate M-cell differentiation by activating non-canonical NF-κB. Thus, both the canonical and non-canonical NF-κB pathways significantly contribute to M-cell development. It should be noted that forced expression of p52/RelB in intestinal organoids effectively induces multiple M-cell markers, such as *Spib, Marcksl1*, and *Anxa1*, except *Gp2* (30). These observations suggest that transcription factors other than NF-κB are required for M-cell maturation.

Among the immature M-cell marker molecules, Spi-B is considered a master regulator of M-cell differentiation (31). Indeed, Spi-B-deficient mice are devoid of GP2^+^ mature M cells. Spi-B deficiency causes a marked loss of expression of *Ccl9, Tnfaip2*, and *Gp2*, whereas early markers, such as *Marksl1* and *Anxa5*, are still expressed (31, 32). Thus, Spi-B is responsible for M-cell maturation rather than early commitment of M-cell fate decision. However, given that forced expression of Spi-B in intestinal epithelial cells does not induce *Gp2* expression (28, 30), Spi-B is necessary, but insufficient, for full maturation of M cells. We recently identified the SRY-related HMG box (Sox) family transcription factor, Sox8, as another master regulator of M-cell maturation (33). Sox8 is induced by RANKL-RelB signaling, concurrent with Spi-B, and directly binds to the *Gp2* promoter region to transactivate gene expression. Sox8-deficient mice display decreased uptake of *Salmonella enterica* serovar Typhimurium (*S*. Typhimurium) as well as nanoparticles into PPs, indicative of a loss of functionally mature M cells. While Sox8-deficient mice largely lack GP2^+^ mature M cells, GP2^−^ Spi-B^+^ immature M cells are generally present in these mice. Interestingly, Spi-B-deficient mice lack *Gp2* expression, although Sox8^+^ cells are present in the FAE. These results imply that both Sox8 and Spi-B are required for the induction of *Gp2* expression *in vivo* (Figure 2B).

Chemokine receptor 6 (CCR6) and its ligand CCL20 may also regulate the number of M cells in PPs. CCL20 is constitutively expressed by FAE, depending on RANKL-RelB signaling (13, 33). CCR6^hi^CD11c^int^ B cells migrate to the SED in response to CCL20 (34). The number of M cells in CCR6-deficient mice is half that in wild-type mice, whereas RANKL expression is not affected in these mice, and adoptive transfer of CCR6^hi^CD11c^int^ B cells from wild-type to CCR6-deficient mice increases the number of M cells (34, 35). These observations suggest that CCR6^hi^CD11c^int^ B cells may play a role in M-cell differentiation, although the underlying mechanism remains unknown.

Colony-stimulating factor 1 receptor (CSF1R)-dependent macrophages also promote the differentiation of epithelial cell linages, including M cells, from Lgr5^+^ stem cells (36). CSF1R signaling controls the proliferation and differentiation of macrophages, and blockade of CSF1R signaling using antibody results in depletion of tissue-resident macrophages in most organs, including the gut (37). CSF1R^+^CD68^+^ macrophages, which express *Wnt4* and *Rspo1*, are in close contact with epithelial cells in the intestinal lamina propria, crypt, and PPs. Depletion of CSF1R^+^CD68^+^ macrophages by blockade of CSF1R signaling causes loss of *Wnt3* expression by Paneth cells, which maintain Lgr5^+^ stem cells. Further, macrophage depletion prominently decreases the expression of M-cell markers, without affecting RANKL expression in SED of PPs. As mentioned above, RANKL supplementation enables intestinal organoids to generate GP2^+^ M cells *in vitro*, even in the absence of CSF1R^+^CD68^+^ macrophages (28). Several components of culture medium for organoids, such as Wnt3 and R-spondin1, may compensate CSF1R^+^ macrophage function.

M cells serve as a portal for potentially hostile microorganisms; therefore, we hypothesize that there should be machinery to regulate the M-cell population and/or maturation to prevent mucosal infection as well as an excessive immune response to the luminal antigens. M cells are scattered throughout FAE in a checkerboard-like pattern and represent only 10–20% of FAE cells of the PP (Figure 1). The numbers and distribution pattern of M cells in FAE may be regulated by Jagged1-Notch signaling (38), the evolutionarily conserved machinery that suppresses the differentiation of secretory cell lineages, namely, goblet, tuft, and enteroendocrine cells, by lateral inhibition (39). Intestinal epithelial cell-specific knockout of *Notch* or its ligands increased the number of *Ulex europaeus* agglutinin-1 (UEA-1)-positive M cells (38).

Interestingly, the number of GP2^+^ mature M cells is significantly lower in FAE of cecal patches than in that of PP (8) (Figure 1), suggesting the existence of suppression mechanisms of M-cell maturation in the cecal patches. The distal GALT, including cecal patches, is continuously exposed to a multitude of commensal bacteria. Collectively, the regulatory mechanisms of the M-cell population and maturation, as well as their role in the mucosal immune system, await further investigations.

M cells are ectopically induced in inflammatory or infectious conditions. M-cell expansion in the colon is observed in inflammatory bowel disease (IBD) patients (40, 41). The differentiation mechanism of “inducible” M cells seems to be different from that of conventional M cells under physiological conditions. For instance, the *S*. Typhimurium-derived type III effector protein SopB causes the transformation of epithelial cells into M cells via activation of Wnt/β-catenin signaling (42). Wnt/β-catenin signaling induces the expression of both RANKL and RANK, and this autocrine activation of RANK signaling may cause the trans-differentiation of enterocytes into M cells. In addition, dextran sulfate sodium (DSS) treatment or *Citrobacter rodentium* infection induces peptidoglycan recognition protein-S (PGRP-S)-positive M cells in the colon via TNF-α/TNFR2 signaling (43, 44). The increase in M cells under inflammatory or infectious conditions may enhance bacterial translocation and worsen the inflammatory response.

## M Cells as A Gateway for Mucosal Antigens

The cellular composition of FAE is distinct from that of the villous epithelium and allows for antigen uptake (45). FAE possesses limited numbers of Paneth and goblet cells, resulting in a thin mucin layer and diminished production of antimicrobial peptides on FAE. This enables luminal antigens (e.g., food antigens, bacteria, and viruses) to readily gain access to M cells. Another characteristic of FAE is the downregulation of IL-22-dependent host-defense molecules because IL-22 signaling is blocked by IL-22 binding protein (IL-22BP/IL-22Ra2), which is abundantly produced in the SED. IL-22BP is a soluble IL-22 receptor that impedes the binding of IL-22 to IL-22Ra1, the membrane-bound IL-22 receptor. Although IL-22BP is dispensable for the development and transcytosis function of M cells, the blockade of IL-22 signaling is essential to limit the barrier function (e.g., antimicrobial production) of FAE (46). This view is corroborated by the observation that IL-22BP deficiency promotes the production of antimicrobial products, which inhibits the uptake of *S*. Typhimurium and *Alcaligenes* into PP. In mice, IL-22BP is highly expressed by MHCII^hi^CD11c^hi^CD11b^+^CD8α^−^ cells in the SED of PPs (46). Another study based on gene expression database demonstrated that monocyte-derived phagocytes highly express *Il22bp* compared to conventional DCs in PPs (47). In addition to PPs, IL-22BP-expressing cells also accumulate in the SED of colonic patches and isolated lymphoid follicles, but not in the lamina propria, suggesting that SED microenvironment may facilitate the expression of IL-22BP (46). In humans, immature monocyte-derived DCs also highly upregulate *IL-22BP* upon stimulation with retinoic acid (48), whereas IL-18 downregulates *IL22BP* expression (49). It should be noted that IL-22 expression by ILC3s in the lymphoid follicles is responsible for preventing bacterial dissemination from PPs to the systemic tissues. Hence, IL-22BP-secreting phagocytes in the SED region establish a firewall to neutralize IL-22 released from the underlying lymphoid follicles. Together, these functional and cellular traits of FAE facilitate antigen sampling via the M cell-dependent pathway.

M cells also possess unique morphological and functional properties. The microvilli on the apical surface of absorptive enterocytes prevent direct adhesion of bacteria to the cell surface. In contrast, M cells have sparse short microvilli (termed “microfolds”) that enable luminal antigens to reach the apical surface. Furthermore, the basolateral membrane of M cells is deeply invaginated, allowing the migration of lymphocytes and DCs into intraepithelial microdomains termed “M-cell pockets” (Figure 2A) (7). The M-cell pockets shorten the distance from the apical surface to the basolateral surface and eventually facilitate antigen transcytosis. Immature DCs that have received antigens from M cells migrate to the interfollicular region rich in naïve T cells to present the antigens. Activated antigen-specific T cells upregulate CXCR5 and migrate to the GC formed in B-cell follicles (3). This T-cell subset, termed “T follicular helper (Tfh) cells,” promotes GC reaction, including class switching (e.g., IgA) and somatic hypermutation. The class-switched IgA^+^ B cells egress GALT and circulate throughout the body. IgA^+^ B cells differentiate into plasma cells and upregulate CCR9 and α4β7 integrin, both of which are required for homing to small intestinal lamina propria (50). Of note, CCR10 is responsible for colon homing (51). Intestinal IgA^+^ plasma cells abundantly secrete dimeric (or polymeric) IgA in the lamina propria. Dimeric IgA binds to the polymeric Ig receptor (pIgR) expressed on the basolateral surface of epithelial cells through a J-chain domain. A recent study demonstrated that marginal zone B and B-1 cell-specific protein (MZB-1) play a critical role in J-chain binding to IgA and secretion of dimeric IgA (52). MZB-1 deficiency suppresses IgA production in the gut, leading to attenuated mucosal barrier function. The complex of IgA homodimer and cleaved pIgR extracellular domain (secretory component) is released in the lumen as S-IgA (53). S-IgA captures luminal antigens to prevent their adhesion on epithelial cells and regulates the balance of intestinal microbiota. Further, the immune complexes composed of S-IgA and luminal antigens are taken up by M cells and subsequently engulfed by macrophages or DCs and induce an immune response (54–56). A recent study revealed that CCR6^+^GL7^−^ antigen-specific B cells are in close contact with M cells in PPs (3). Surprisingly, these B cells receive antigens directly from M cells in a DC-independent manner. The antigen-bound B cells then migrate from the SED to the GC. However, the significance of the M-cell–B-cell axis in the IgA response remains to be elucidated.

While M cells play a vital role in mucosal immunosurveillance, active antigen transport by M cells potentially provides vulnerable gateways in the robust epithelial barrier. Indeed, *Salmonella typhi* and *Shigella flexneri* gain entry into the body via M cells (57) (Table 1). Further, M cells transport several viruses, such as human influenza virus, norovirus, and reovirus (58, 59). Thus, various pathogenic bacteria, viruses, toxins, and prions exploit M cells as a portal to bypass the epithelial barrier and establish systemic infection (7), implying that M cell-dependent antigen uptake could be a double-edged sword in the context of mucosal infection and host defense.

## Molecular Insights Into Antigen Uptake by M Cells

M cells express a variety of receptors for antigen uptake (Table 1) (60). Among these, GP2, a glycosylphosphatidylinositol (GPI)-anchored protein, was originally identified as a secretory protein expressed in the pancreas (61). We previously reported that GP2 is expressed at the apical surface of M cells and functions as an uptake receptor for type-I-piliated bacteria by binding FimH, a component of type I pili (9). GP2-deficient mice exhibit attenuated uptake activity of type-I-piliated bacteria, such as *Escherichia coli* and *S*. Typhimurium. Intriguingly, GP2 also serves as an uptake receptor for botulinum toxin A complex (62). GP2 binds to the nontoxic hemagglutinin (HA) domain of botulinum neurotoxin complexes. Because GP2 expression by M cells is conserved among human and mice, this protein is considered a universal marker of M cells across species. M cells also express the other GPI-anchored protein, cellular prion protein (PrP^C^), which serves as an invasive receptor for *Brucella abortus*, causing brucellosis (63). PrP^C^ deficiency results in reduced uptake of *B. abortus* in PPs. PrP^C^ is considered to bind with heat shock protein (Hsp) 60 on *B. abortus* (64). Moreover, M cells may function as a portal for exogenous scrapie-type prion protein (PrP^Sc^), the infectious isoform of the prion protein. This fact suggests that the M cell-dependent pathway may be implicated in the pathogenesis of scrapie and other prion diseases such as variant Creutzfeldt–Jakob disease in humans (65). β1 integrin is expressed in the basolateral plasma membrane of epithelial cells to function as a receptor for extracellular matrix and as a cell adhesion molecule; however, it is also expressed at the apical surface of M cells and serves as an uptake receptor for *Yersinia enterocolitica* in these cells (66). To bind with conventional ligands (e.g., fibronectin, collagen, and laminin), β1 integrin has to be activated. In M cells, β1 integrin activation is mediated by allograft inflammatory factor 1 (Aif1) (67). Although Aif1-deficient mice regularly express β1 integrin in M cells, Aif1 deficiency diminishes the internalization of *Y. enterocolitica* into PP due to inactivation of β1 integrin. Aif1 deficiency also impairs the uptake of nanoparticles by M cells, suggesting that this molecule may also play a role in receptor-independent transcytosis. In macrophages, Aif1 promotes phagocytosis by inducing actin remodeling through the activation of a small GTPase, RAS-related C3 botulinum toxin substrate 1 (Rac1) (68). Activated Rac1 remodels actin filaments to promote membrane ruffling and vesicular transport. Hence, Aif1 may also cause actin remodeling by activating Rac1 to facilitate transcytosis of luminal macromolecules.

A unique DC subset characterized by high expression of lysozyme (LysoDC) takes up luminal antigens by protruding their dendrites (or migrating to the lumen) through transcellular pores in UEA-1-positive M cells (69). F-actin and cell adhesion molecules are strongly recruited to the dendrites of LysoDC to engulf luminal nanoparticles and *S*. Typhimurium before retracting back to the SED. Another study reported that small vesicles are released from the basolateral pocket of M cells and are subsequently taken up by CX_3_CR1^+^CD11b^+^CD11c^+^ DCs in the SED (70). In this study, the authors employed PGRP-S-dsRed mice to label M cells and demonstrated that dsRed-positive vesicles were colocalized with transcytosed bacteria, such as *Staphylococcus aureus*. M cell-derived vesicles are constitutively released, and both gram-positive bacteria and TLR2 agonist increase the number of vesicles. Thus, the mechanism of antigen uptake by M cells is complex and remains enigmatic. Further investigation is required to clarify the biological significance of LysoDC and the M cell-derived vesicle-dependent antigen uptake system.

## Local and Systemic Immune Responses Induced by M-Cell-Dependent Antigen Uptake

Multiple studies have demonstrated that antigen uptake by M cells is critical for antigen-specific immune responses. Oral administration of bacteria (e.g., *S*. Typhimurium and *Y. enterocolitica*) or soluble antigens (e.g., ferritin and cholera toxin) induces antigen-specific S-IgA, depending on M cells (9, 13, 31, 67, 71). Epithelial RANK-deficient (RANK^Δ*IEC*^) mice lacking both mature and immature M cells display antigen uptake defect and decreased GC maturation in PPs (71). Further, RANK^Δ*IEC*^ mice showed a reduced number of IgA^+^ B cells in the lamina propria and attenuated S-IgA production in the gut (13, 71).

Sox8-deficient mice lack mature GP2^+^ M cells, whereas immature M cells are adequately present (33). Similar to RANK^ΔIEC^ mice, Sox8 deficiency reduces IgA production in the gut, albeit to a lesser extent. This abnormality was observed around the weaning period. Later, fecal IgA levels gradually increased and reached a normal level in adulthood, although the number of mature M cells remained substantially lower in these mice. These observations suggest that mature M cells with high antigen uptake capability may be vital for the establishment of the gut immune system until weaning, during which mice are susceptible to infection because of the decline in breast milk-derived maternal antibodies.

Furthermore, the absence of M cells due to intestinal epithelium-specific deletion of NIK decreases the induction of serum IgA as well as serum IL-17 in DSS-induced colitis and a polymicrobial sepsis model (29). Loss of IL-17 or IgA also increases the susceptibility to these diseases, suggesting that M cell-dependent systemic IL-17 and IgA responses protect against colitis and sepsis. In contrast, constitutive activation of NIK signaling causes ectopic M-cell expansion in the colon as well as high IL-17 production and exacerbates DSS-induced colitis (29). In addition, we previously reported that M cell-dependent antigen uptake contributes to the induction of a systemic antigen-specific IgG response (9). These observations exemplify that antigen uptake by M cells plays crucial roles not only in mucosal but also in systemic immune responses.

## M Cells in Tertiary Lymphoid Structures in Systemic Autoimmune Diseases

Autoimmune diseases are often associated with ectopic formation of lymphoid tissues in target organs, such as the kidneys, heart, pancreas, synovium, salivary glands, and lungs, which are not embryologically programmed to form lymphoid tissue (72). Such postnatally induced lymphoid tissue-like structures are classified as tertiary lymphoid structures (TLSs) (73). Secondary lymphoid tissues like PP, lymph nodes, and the spleen are highly organized with architectural domains that facilitate cellular dialogue between antigen-presenting cells and lymphocytes to promote B- and T-cell activation, selection, and differentiation, ultimately increasing the efficiency of the immune response. TLSs share some morphological and cellular features with secondary lymphoid tissues, such as segregated T- and B-cell zones, follicular DC networks, and high endothelial venules, but they lack a stable capsulated structure covering lymphoid follicles. TLSs may establish a microenvironment to facilitate an adaptive immune response to local antigens.

Inducible bronchus-associated lymphoid tissue (iBALT) is a TLS that is formed in the lungs and respiratory tracts during chronic inflammation, allergy, or infection (74). iBALT is also frequently found in interstitial lung disease associated with systemic autoimmune conditions, such as rheumatoid arthritis (RA) and scleroderma (74). This lymphoid tissue preferentially arises in close proximity to tracheal and/or bronchial epithelia but does not possess the archetypal FAE nor the SED region where antigen-presenting cells accumulate. M cells have been reported in iBALT of some species based on morphological features and lectin reactivity (75–77), although the molecular features of these cells remain obscure. We recently characterized M cells in iBALT by detecting M-cell signature molecules in four mouse models of iBALT formation (78). We detected mature M cells in iBALT-associated epithelium under spontaneous autoimmune conditions in nonobese diabetic (NOD) mice based on upregulation of GP2, Tnfaip2, and RANK. The airway M cells may mediate respiratory infection with *Streptococcus pyogenes* and *Mycobacterium tuberculosis* (79, 80). Therefore, it is interesting to speculate that M cells in iBALT may participate in the pathogenesis of interstitial lung diseases.

## Contribution of Intestinal Microbiota to Autoimmune Diseases

Autoimmune diseases are caused by an inappropriate immune response against own tissues and cell components, resulting in not only local tissue-specific but also systemic inflammation, leading to tissue damage. The incidence of some autoimmune diseases, represented by multiple sclerosis (MS) and type 1 diabetes mellitus (T1D), has increased globally, especially in economically developed countries (17, 81, 82). Earlier diagnosis and improved access to the hospital only partly account for the increased incidence in these countries. Changes in lifestyle, including dietary habits, exposure to environmental agents, a decrease in infectious diseases, and vaccination, have been considered predisposing factors for autoimmune diseases (83, 84). Indeed, multiple lines of evidence have suggested an interaction between environmental and genetic factors in disease susceptibility as well as disease phenotypes. The high concordance ratio between monozygotic twins when compared to dizygotic twins supports the idea that genetic factors play a significant role in the pathogenesis of some autoimmune diseases, such as T1D and Crohn's disease (CD) (85–87), although there remains discordance in disease development even between monozygotic twins. Moreover, other systemic autoimmune diseases, such as RA and MS, exhibit a lower concordance rate in monozygotic twins (88–91), indicating that environmental factors also significantly contribute to the pathogenesis of autoimmune diseases. In recent years, compelling evidence implied a potential involvement of mucosal immune dysregulation associated with intestinal dysbiosis in disease onset. Therefore, the intestinal microbiota has emerged as a critical environmental factor in the development of autoimmune diseases (14–18, 84, 92). Considering that PPs serve as an inductive site for mucosal immune responses, PPs may facilitate the development of autoimmune disease in several autoimmune models. For instance, segmented filamentous bacteria (SFB; also termed *Candidatus Savagella*) induces atherogenic Tfh cells in PPs, leading to the development of autoimmune arthritis (93). In contrast, oral immune tolerance to autoantigens is established in PPs (94). Thus, it remains debatable whether PPs are an inductive or regulatory site for autoimmune diseases. In the following four sections, we will focus on the causal relationships between commensal microbiota, the mucosal immune system, and pathogenesis of both organ-specific and systemic autoimmune diseases. In addition, the potential contribution of M cell-dependent transport of intestinal microbes to GALT will be explored.

## GP2 Autoantibody and M-Cell Expansion in IBD

CD is an IBD associated with chronic inflammation throughout the gastrointestinal tract (95). The pathogenesis of CD remains to be fully elucidated; however, a combination of genetic background, dietary factors, and intestinal dysbiosis affect the susceptibility to this disease. Dysregulation of both innate and adaptive immune systems is evident in CD patients. Further, immune tolerance to autoantigens is thought to be compromised, and ~40% of CD patients produce pancreatic autoantibodies (96). Eventually, a fraction of CD patients develop pancreatitis as a complication. Interestingly, pancreatic autoantibodies from CD patients recognize GP2 as an antigen (97), because GP2 is abundantly expressed by acinar cells of the pancreas and secreted into the intestinal lumen with pancreatic juice.

**Figure 3:**
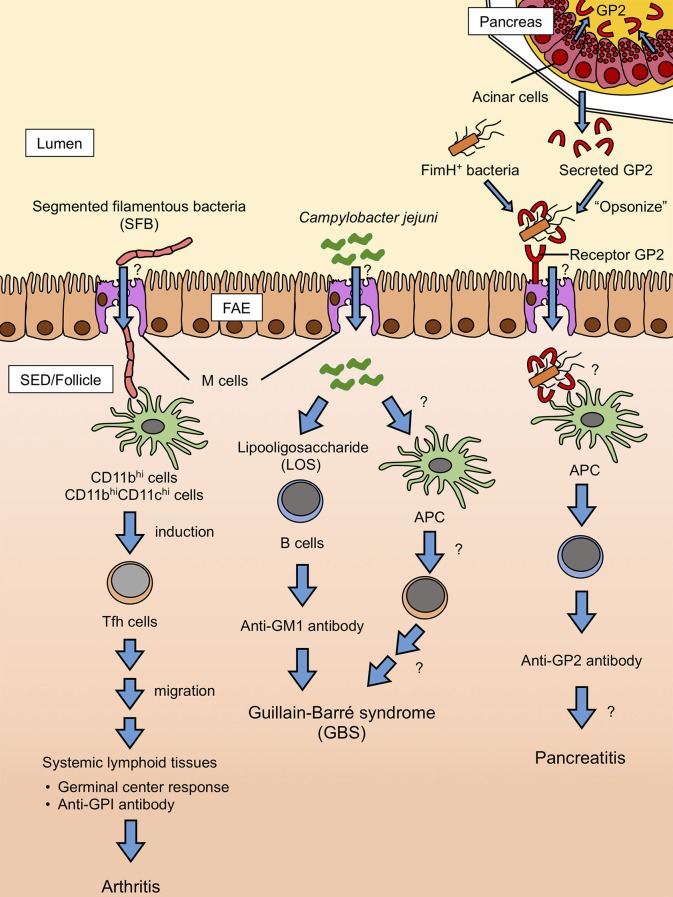
Hypothetical model of autoantibody production mediated by M cells. SFB is delivered to CD11b^hi^ and CD11b^hi^ CD11c^hi^ cell subsets in PP via M cell-mediated transcytosis. The M cells engulf SFB, which induces the differentiation of Tfh cells. Tfh cells egress from the PP and migrate to the systemic lymphoid tissues where autoimmune responses predominantly occur, leading to the production of autoantibodies to glucose-6-phosphate isomerase and thus, exacerbation of arthritis. It is unlikely that SFB induces Tfh differentiation by molecularly mimicking the glucose-6-phosphate isomerase antigen. *C. jejuni* also can be taken up by M cells. B cells directly recognize *C. jejuni* LOS, probably through Toll-like receptor 4 (TLR4) engagement, and produce LOS-specific antibodies. LOS is structurally homologous to GM1 ganglioside, which can lead to the production of anti-GM1 antibodies. Anti-GM1 antibodies are associated with some forms of GBS. Likely, *C. jejuni*-derived protein antigens are also involved in the pathogenesis of GBS through the activation of Tfh and B cells. Pancreatic acinar cells secrete GP2 to the lumen, where it binds FimH-positive bacteria. FimH-positive bacteria with GP2 are taken up by M cells via receptor GP2. Antigen-presenting cells (APCs) recognize GP2 on the surfaces of bacteria and start producing anti-GP2 autoantibodies. Anti-GP2 autoantibodies may target GP2-expressing pancreatic cells to cause pancreatitis.

The biological significance of secretory GP2 remains uncertain. Like membrane-bound GP2 on the M-cell surface, secretory GP2 binds to FimH of gram-negative bacteria. Further, GP2 self-polymerizes into high-molecular aggregates (98). We speculate that secretory GP2 in the intestinal lumen may bind with certain food-borne bacteria to form aggregates, which should prohibit epithelial adhesion and invasion of the bacteria. Indeed, electron micrography revealed that intestinal bacteria are surrounded by secretory GP2 (99). Thus, FimH^+^ bacteria “opsonized” by secretory GP2 are taken up by M cells via receptor GP2 and may induce autoantibodies against GP2 (Figure 3) (96). Furthermore, dysbiosis and/or dysfunction of immune tolerance under inflammatory conditions may promote GP2 autoantibody production in CD patients. Alternatively, but not mutually exclusive, inflammatory stimuli may cause M-cell population expansion in the intestine. Indeed, the number of M cells in the colon is increased in both human CD patients and mice with DSS-induced colitis (40, 44). Enhanced M cell-dependent antigen uptake might similarly cause an autoimmune response to GP2 in CD patients. However, further investigation is required to determine whether anti-GP2 autoantibodies are a result of inflammation or are implicated in the pathogenesis of complications such as pancreatitis.

Ulcerative colitis (UC) is another subtype of IBD, in which the colonic mucosa develops ulcerous lesions. The pathogenesis of UC is uncertain; however, like CD patients, UC patients exhibit an aberrant immune response to intestinal microbiota, leading to chronic inflammation (100). Single-cell transcriptome analysis of colon biopsies from UC patients revealed that M-like cells that express *SPIB* and *SOX8* are expanded in patients compared with healthy subjects (41). Further, receptor-ligand analysis to construct a putative cell–cell interaction network indicated that the M-like cells serve as a central node in the network during inflammation. Notably, the M-like cells highly upregulate IBD-susceptible genes (e.g., *CCL20, NR5A2, JAK2, PTGER4, SH2B3*, and *AHR*) as indicated by a genome-wide association study (41). These observations support a potential role of M-like cells in IBD development.

## Intestinal Microbiota and T1D Development

T1D is an organ-specific autoimmune disease characterized by destruction of β-cells within the pancreatic islets, leading to insulin deprivation. The incidence of T1D increases annually by 1.8% worldwide (101, 102). T1D consists of two subtypes; with or without autoimmune response. The autoimmune type, which is characterized by the production of autoantibodies, such as anti-insulin autoantibody, anti-protein tyrosine phosphatase-like protein, and anti-zinc transporter eight antibodies, is dominant. The concordance rate for T1D in monozygotic twins is ~50%, although it varies depending on age at diagnosis. For instance, the concordance rate in very young-onset (younger than 5 years at diagnosis) T1D monozygotic twins is up to 85%, whereas that in adult-onset T1D monozygotic twins is remarkably lower, underscoring the impact of environmental factors on disease onset later in life (103).

NOD mice spontaneously develop T1D with pathological symptoms similar to those of human T1D. Under specific pathogen-free (SPF) conditions, female NOD mice are 4.4-fold more likely to develop T1D than male mice are. However, such gender difference in T1D susceptibility largely disappears under germ-free (GF) conditions (104), implying that the commensal microbiota causes gender bias in NOD mice. Treatment with antibiotics, such as tylosin tartrate, accelerates T1D development in NOD (105, 106). In contrast, treatment of pregnant NOD mice with an antibiotic cocktail protects their offspring from T1D development by an unknown mechanism (107). These studies support the significance of the commensal microbiota in T1D development, and particular bacterial species may influence T1D susceptibility. In NOD mice, insulitis due to the destruction of pancreatic β-cells precedes T1D. Insulitis is caused by autoreactive Th1 cells that produce IFN-γ. NOD mice harboring SFB in the ileum develops insulitis at similar extent with SFB-negative NOD mice. Nevertheless, the subsequent development of diabetes was inhibited in the presence of SFB (108). Because SFB is a potent inducer of Th17 cells, the protective effect of SFB on diabetes may be attributed to the suppression of the Th1 response as a counterreaction to enhanced Th17 responses. Given that these SFB-induced Th17 responses are mainly induced in PPs and isolated lymphoid follicles (109), PPs may be the primary site for the autoimmune response in insulitis development in NOD mice. Moreover, SFB facilitates the expansion of Tfh cells in ileal PPs, as described in the following section.

## Dysbiosis as A Predisposing Factor of RA

RA is a chronic autoimmune disease characterized mainly by synovial inflammation, destruction of cartilage bone of multiple joints, and disability. The incidence of RA is 0.5–1.0% worldwide (110). RA is divided into seropositive and seronegative types, with the former being the most common. Seropositive RA patients are characterized by enhanced serum autoantibodies, i.e., rheumatoid factor (anti-Fc potion of IgG) and anti-cyclic citrullinated peptide antibody. Environmental factors are likely to play a critical role in RA development in genetically susceptible individuals. Although specific environmental factors that affect RA development and progression remain to be defined, numerous clinical studies have suggested that the intestinal microbiota plays a key role in the etiology of RA. This view is supported by findings in animal studies using experimental RA models, such as IL-1 receptor antagonist-deficient (*Il1rn*^−/−^) mice, SKG mice, and K/BxN mice; all of these RA models failed to develop arthritis under a GF environment (93, 111, 112). Furthermore, SKG mice transplanted with microbiota from new-onset, untreated RA patients, which is dominated by *Prevotellaceae*, exhibit an increase in Th17 cells in the gut and spontaneously develop severer arthritis than those transplanted with microbiota from healthy subjects (112). This observation underscores the significance of intestinal dysbiosis in the development of autoimmune arthritis.

In some seropositive RA patients, the rheumatoid factor of IgA isotype, as well as anti-cyclic citrullinated peptide (CCP) IgA antibodies, were detected before the onset of the disease (113, 114). Considering that the IgA response primarily takes place in GALT and IgA^+^ plasma cells are mainly located in the intestinal lamina propria, the mucosal tissue may initiate autoimmune responses in RA in the early phase of disease development. Ileal colonization by SFB in K/BxN mice markedly induced the differentiation of Tfh cells in PP before the development of arthritis (93). SFB-induced Tfh cells egress from PP to systemic lymphoid tissues, such as the spleen and joint-draining lymph nodes, to promote the generation of autoantibodies. Importantly, depletion of PP confers resistance to arthritis development in K/BxN mice (93). Although SFB is closely associated with the FAE surface of ileal PP, it remains unknown whether this bacterial species is taken up by M cells (Figure 3). However, these observations illustrate that GALT serves as an initiator and/or enhancer of autoantibody production in the RA model. It is interesting to extend this view to other autoantibody-dependent diseases, such as systemic lupus erythematosus and Sjogren's syndrome.

## Intestinal Dysbiosis Associated With Ms

MS is an autoimmunity-mediated demyelinating disease that causes injury to the central nervous system (84). MS patients develop chronic disorders such as cognitive and motor control impairments. Dysbiosis of the gut microbiota has been observed in MS patients (115, 116). MS-associated dysbiosis is characterized by an overrepresentation of *Acinetobacter calcoaceticus, Akkermansia muciniphila* (117), and *Methanobrevibacter*, and an underrepresentation of *Butyricimonas* (116). Notably, bacterial extracts of *A. calcoaceticus* suppress the differentiation of Treg cells in peripheral blood mononuclear cells derived from healthy subjects. Conversely, components of both *A. calcoaceticus* and *A. muciniphilas* promote the differentiation of Th1 cells (117).

Experimental autoimmune encephalomyelitis (EAE) is a commonly used animal model of MS. EAE is induced by immunization with myelin components, such as myelin basic protein (MBP) and myelin oligodendrocyte glycoprotein (MOG) peptides. Like RA models, GF mice are resistant to EAE development. However, transplantation of gut microbiota from SPF to GF mice causes EAE symptoms (109). In contrast, several commensals exert a protective effect against EAE. For example, polysaccharide A from *Bacteroides fragilis* induces IL-10-producing Treg cells that alleviate the development of EAE (118). Analogous to arthritic K/BxN mice, SFB mono-colonization is sufficient to initiate EAE development by promoting Th17 responses (109). Thus, both clinical observations and animal studies have indicated that the gut microbiota is a key determinant of MS development. However, it remains largely unknown how these bacteria influence the immune system across the epithelial barrier. Th17 cells are increased and Treg cells reduced in the intestinal lamina propria, PPs, and mesenteric lymph nodes in mice with EAE, supporting a link between the alteration of gut immune responses and EAE development (119). Furthermore, oral administration of MBP to EAE mice causes upregulation of monocyte chemoattractant protein 1 (MCP-1) in the small intestine to recruit autoreactive T cells to PPs, where they undergo apoptotic cell death (94). MBP feeding for seven days before immunization protects mice from acute EAE. This suggests that PPs may also contribute to the induction of oral tolerance to MBP. Thus, the targeting of autoantigens to PP M cells could be a promising strategy to suppress autoimmune reaction.

## Infectious Agents and GBS

GBS is an acute inflammatory nervous disease that causes numbness in the legs and arms (120). Although the cause of GBS is not yet fully elucidated, the autoimmune response has been implicated in the pathogenesis. Autoantibodies against gangliosides embedded in the lipid raft of the plasma membrane are detected in ~60% of GBS patients. The anti-ganglioside autoantibodies comprise various isotypes: IgG1, IgG3, IgA, and IgM (121). These autoantibodies injure the myelin sheaths of the peripheral nerves. Notably, GBS is known to develop after infection or vaccination (122). There is also a causal relationship between GBS and several infectious microbes, such as cytomegalovirus, Epstein-Barr virus, *Mycoplasma*, and *Campylobacter* (123). In particular, *Campylobacter jejuni* is a major pathogen of antecedent infections identified in 20–30% of GBS cases. Serum anti-ganglioside IgA in GBS patients is closely correlated with anti-*C. jejuni* IgG (124). The induction of anti-GM1 ganglioside autoantibody is thought to be caused by the structural homology between GM1 and lipo-oligosaccharide (LOS) of *C. jejuni* (125–127). However, LOS of *Campylobacter coli* does not evoke GBS, although it shows homology to GM1 (128). Furthermore, anti-GM1 antibodies from GBS patients fail to cross-react with LOS from *C. coli*. Thus, subtle molecular structural differences in LOS between species may determine its antigenicity which induces autoantibody production (128). How immune cells recognize *C. jejuni*-derived LOS is currently an open question. Interestingly, an early study indicated that *C. jejuni* selectively adheres to M cells and is transported into follicles of PP in rabbits (129). Besides, anti-GM1 IgA is detected in the serum of GBS patients after infection by *C. jejuni* (124). Therefore, in some cases of GBS, M cell-dependent uptake of *C. jejuni* may trigger the generation of antibodies cross-reacting to GM1, which eventually leads to the development of GBS (Figure 3). Further investigations will be needed to clarify the underlying pathological mechanism of autoantibody generation in GALT.

## Conclusions and Perspectives

Accumulating studies have disclosed not only correlational, but also causal relationships between intestinal microbiota and autoimmune diseases. Nevertheless, how intestinal microbes induce autoimmune responses remains to be elucidated. In certain autoimmune diseases, GALTs, such as PPs, may contribute to the development of autoimmune Th17 cells as well as autoantibody production. Therefore, we consider that PPs may serve as not only an inductive site for the mucosal immune response, but also an amplifier of the autoimmune response. M cell-dependent bacterial transport to PPs may initiate such autoimmune responses. Further research is needed to provide proof of this concept. Future studies of M cell-deficient or PP-null mice should provide new insights in the pathogenesis of several autoimmune diseases. Further exploration of specific bacterial species responsible for the susceptibility to various autoimmune diseases would lead to the development of new therapeutic strategies for autoimmune disorders by targeting intestinal bacteria.

## Author Contributions

NK and DT mainly wrote the manuscript and the figures. SK wrote a part of the manuscript. SK and ST performed the immunofluorescent staining. KH edited the manuscript and obtained funding.

### Conflict of Interest

The authors declare that the research was conducted in the absence of any commercial or financial relationships that could be construed as a potential conflict of interest.
